# Amniotic Fluid or Its Fatty Acids Produce Actions Similar to Diazepam on Lateral Septal Neurons Firing Rate

**DOI:** 10.1155/2013/534807

**Published:** 2013-06-24

**Authors:** Ana G. Gutiérrez-García, Carlos M. Contreras, Diana Idania Vásquez-Hernández

**Affiliations:** ^1^Laboratorio de Neurofarmacología, Instituto de Neuroetología, Universidad Veracruzana, Avenue Dr. Luis Castelazo S/N Col. Industrial Las Ánimas, 91190 Xalapa, VER, Mexico; ^2^Facultad de Psicología, Universidad Veracruzana, Manantial San Cristóbal-Xalapa 2000, 91097 Xalapa, VER, Mexico; ^3^Unidad Periférica Xalapa, Instituto de Investigaciones Biomédicas, Universidad Nacional Autónoma de México, 91190 Xalapa, VER, Mexico

## Abstract

Human amniotic fluid (AF) contains eight fatty acids (FATs), and both produce anxiolytic-like effects in adult rats and appetitive responses in human newborns. The medial amygdala and lateral septal nucleus function are related to social behavior, but the action of AF or its FATs in this circuit is known. We obtained 267 single-unit extracellular recordings in Wistar rats treated with vehicle (1 mL, s.c.; *n* = 12), human AF (1 mL, s.c.; *n* = 12), a FAT mixture (1 mL, s.c.; *n* = 13), diazepam (1 mg/kg, i.p.; *n* = 11), and fluoxetine (1 mg/kg, p.o.; *n* = 12). Compared with the vehicle group, the spontaneous septal firing rate in the AF, FAT mixture, and diazepam groups was the lowest and in the fluoxetine group the highest. Cumulative peristimulus histograms indicated that the significant change in septal firing occurred only in the AF and FAT mixture groups and exclusively in those neurons that increased their firing rate during amygdala stimulation. We conclude that human AF and its FATs produce actions comparable to anxiolytic drugs and are able to modify the responsivity of a circuit involved in social behavior, suggesting facilitation of social recognition processes by maternal-fetal fluids.

## 1. Introduction

Newborn mammals, including humans, exhibit agitation and emit vocalizations when placed in an unfamiliar environment, but they calm down when they return to their nest or remain in close proximity to their mother [1, 2], an action likely related to odors that come from maternal fluids, such as amniotic fluid (AF), colostrum, and milk [3–5]. These observations open the possibility that the calming effect is an anxiolytic property of maternal fluids.

We recently reported that human AF and its fatty acids (FATs) produce anxiolytic-like effects in adult Wistar rats subjected to validated models of experimental anxiety [6]. In such a case, the fetus seemingly develops and grows in a comfortable medium that provides a protective anxiolytic action through an unknown process. Maternal fluids, such as AF, colostrum, and milk, also produce orientating movements in some mammals [7–9], which include human newborns [10–14]. These three maternal-infant fluids that contain FATs are quite different in pigs [15] and humans [6]. Amniotic fluid or an artificial mixture of its FATs produces orientating-feeding responses in human newborns [16], illustrating early learning [17] through an unknown neural process.

The lateral septal nucleus contains *γ*-aminobutyric acid (GABA) and serotonin (5-hydroxytryptamine [5-HT]) terminals [18] and is part of a functional network related to social behavior processing [19]. Septal neurons increase their firing rate after the administration of fluoxetine [20], among other clinically effective antidepressant drugs [21], and decrease their firing rate after the administration of benzodiazepines [22, 23]. Anxiolytic GABAergic drugs produce a low septal firing rate, whereas serotonergic drugs with anxiolytic actions produce a high neuronal firing rate in the lateral septal nucleus. Diazepam exerts anxiolytic effects through its well-known action on GABA_A_ receptors [24]. Fluoxetine is an antidepressant that selectively inhibits serotonin reuptake to also produce anxiolytic effects [25]. Therefore, these two drugs may be useful tools for exploring the anxiolytic effects of AF or its FATs on neuronal activity.

Any possible action of AF and its FATs on the neuronal activity of lateral septal neurons has not been explored. The aim of the present study was to compare the effects of AF and its FATs against diazepam and fluoxetine on the neuronal firing rate of lateral septal neurons in response to medial amygdala stimulation.

## 2. Materials and Methods

### 2.1. Ethics

For the human samples, we strictly followed international principles of confidentiality and healthcare, such as the Declaration of Helsinki, and all of the procedures in rats followed the principles of animal care based on the Guide for the Care and Use of Laboratory Animals [26]. Both protocols received authorization from the Biomedical Research Institute Ethical Committee (National Autonomous University of México).

### 2.2. Human Samples

All of the volunteers were given a detailed explanation of the purpose and risks of the study and signed an informed consent prior to inclusion in the study. Volunteers were invited to participate only as donors of AF, with no risk to the mother or baby. Both the mother and newborn were in optimal health, reflected by a general clinical evaluation of the mothers and proper scales for newborns. Amniotic fluid was obtained under sterile conditions by an obstetrics surgeon. For vaginal deliveries, after the amniotic membrane ruptured, approximately 5 mL of AF was collected in a sterile receptacle. For caesarean deliveries, the surgeon collected the AF with a sterile syringe (5 mL, 23 gauge). A total sample of approximately 50 mL of AF was collected from 11 healthy volunteers (15–30 years old; 37–42 weeks gestational age; first gestation, *n* = 4; vaginal delivery, *n* = 5).

### 2.3. Experimental Groups

Given that spontaneous lateral septal neuronal firing varies during the estrous cycle in Wistar rats [27], the present study included 60 male rats that were randomly assigned to five experimental groups. The single administration or last administration of the treatments preceded anesthesia for the surgical procedures by 30 min. After another 60 min, the first single-unit extracellular recording was obtained.

Fresh human AF (1 mL/rat, *n* = 12) was injected subcutaneously after being filtered with filter paper (no. 4, 110 mm diameter; Whatman International, Maidstone, UK).

A fatty acid mixture (1 mL/rat, *n* = 13) was injected subcutaneously. The selection of the concentrations of the FATs contained in the artificial FAT mixture was based on previous reports [6, 16, 17]. The FAT mixture group received a mixture of lauric acid (0.4 mg), myristic acid (3.0 mg), palmitic acid (15.3 mg), palmitoleic acid (7.1 mg), stearic acid (3.7 mg), oleic acid (8.0 mg), elaidic acid (1.5 mg), and linoleic acid (4.4 mg) dissolved in 100 mL of vehicle (96% propylene glycol and 4% ethanol) at a temperature of 37–40°C. All of the FATs were analytical grade and obtained from Sigma-Aldrich (St. Louis, MO, USA).

The study included one vehicle group (*n* = 12) that received a single subcutaneous injection of the FAT mixture solvent. One active drug control group (*n* = 11) received a single dose of diazepam (Valium, 1 mg/kg; Roche, Toluca, México) dissolved in isotonic saline (0.9%) and injected intraperitoneally in a volume of 0.3 mL/rat. A second active drug control group (*n* = 12) received fluoxetine (Prozac, 1 mg/kg; Eli Lilly de México, Tlalpan, México) dissolved in isotonic saline (0.9%) and administered orally in a volume of 1.0 mL/kg daily for 21 days [20].

### 2.4. Animals and Housing

Wistar rats from a local strain initially supplied by Harlan (México City, México), approximately 3 months old and weighing 250–300 g, were included in the study after being housed in local facilities at a mean temperature of 25°C with a 12 h/12 h light/dark cycle (lights on 7:00 a.m.). They were housed five to six rats per cage in acrylic boxes (44 cm width × 30 cm length × 20 cm height) with free access to food (Teklad Lab Animal Diets, Harlan) and purified water. All of the experimental procedures were performed during the light period, beginning at 9:00 a.m.

### 2.5. Single-Unit Extracellular Recordings

#### 2.5.1. Stereotaxic Surgery

Thirty minutes after the last injection of any treatment, the rats were profoundly anesthetized with ethyl carbamate (1 g/kg urethane, intraperitoneally; Sigma Chemical, St. Louis, MO, USA), and their head was fixed in a stereotaxic frame (Stoelting, Wood dale, IL, USA) to proceed with surgery. Cardiac pulse and a parietal cortical surface electroencephalogram were continuously monitored on a polygraph (GRASS 79, Grass Instruments, Quincy, MA, USA). During recording, we added one-tenth of the initial dose of urethane upon detecting signs of alertness, such as respiratory acceleration, movements of the vibrissae, or blinking, or sudden changes in cardiac pulse. A midline incision uncovered the skull. Through a small trephination, we lowered a glass micropipette filled with 1 M NaCl (4-5 MΩ) using a hydraulic micromanipulator (Trent Wells, South Gate, CA, USA) toward the lateral septal nucleus (coordinates: anterior/posterior, 0.2 mm; lateral, 0.5 mm; dorsal/ventral, from −3.0 to −5.0 mm) [28]. Another trephination was made at coordinates that correspond to the medial amygdala (anterior/posterior, 2.8 mm; lateral, 3.3 mm; ventral, −8.6 mm), where a stainless-steel bipolar electrode was placed (~100 kΩ resistance, 1 mm insulation uncovered at the inner tip, 100 *μ*m diameter).

#### 2.5.2. Single-Unit Extracellular Recordings

The micropipette signal was connected in series to a 7P511L Grass amplifier (Quincy, MA, USA; bandwidth pass filters: 300 Hz–3 KHz) and an oscilloscope (model 5111A, Tektronix, Beaverton, OR, USA) that received a filtered signal free from background noise through a window discriminator and in parallel to an audio amplifier. The absence of sudden changes in the amplitude of the firing rate over 300 s verified a stable recording. Afterward, each spike detected by the amplifier was fed to a Grass S88 stimulator (Quincy, MA, USA) that delivered a spike-corresponding square pulse of constant amplitude and duration (4 V, 0.6 ms) directed to a CED 1401 interface system (Cambridge Electronic Design, Cambridge, UK). The basal activity of lateral septal neurons was recorded for 1 min, followed by 1 min of amygdala stimulation (20 stimuli, monophasic pulses, 0.3 Hz, 0.6 ms duration). The signals were processed (bin time, 1 ms) by Spike2 software, version 5.20, that delivered the mean and standard error of the firing rate (c/10 s) of spontaneous activity and generated 1000 ms base peristimulus histograms.

#### 2.5.3. Histological Analysis

To mark the last recorded point in the single-unit extracellular recording of the lateral septal nuclei, we first passed a direct current (1 min each polarity) through the recording micropipette. The stimulating electrodes were marked with electrolytic lesions (0.5 mA for 30 s for each polarity). Afterward, the rats received a lethal overdose of pentobarbital and were intracardially perfused with 200 mL of 0.9% saline solution, followed by 200 mL of 30% formaldehyde. The brains were removed and postfixed with 30% formaldehyde for 72 h. The tissues were cryoprotected with 30% sucrose for 24 h, frozen at −20°C, cut into 40 *μ*m-thick sections with a cryocut microtome (Leica-Jung, Nussloch, Germany), and dyed using the Nissl technique to reconstruct the path followed by the micropipette and stimulation electrodes with the aid of stereotaxic coordinates [28].

### 2.6. Data Collection and Statistical Analysis

We analyzed (SigmaStat version 3.5) the baseline lateral septal nucleus firing rate under basal conditions for 1 min before amygdala stimulation. One-way analysis of variance (ANOVA) was used to compare the overall effects of the treatments (vehicle, AF, FAT mixture, diazepam, and fluoxetine), followed by the Student-Neuman-Keuls (SNK) *post hoc* test. Values of *P* ≤ 0.05 were considered statistically significant.

Based on the peristimulus histograms (1000 ms), we formed three groups of septal neurons: neurons that increased (↑ cells) or decreased (↓ cells) their firing rate or had no response (Ø cells) to amygdala stimulation. The criterion of change was based on a poststimulus firing rate difference that was higher or lower than the mean value of the prestimulus firing rate ±1 standard deviation. After classifying neuronal activity, a complete database was constructed with all of the recorded neurons. Therefore, the cumulative peristimulus histograms and their corresponding statistics included all of the recorded neurons, classified according to their response to amygdala stimulation. For the analysis of these data, the prestimulus data were considered the basal firing rate, and the percentage of change in neuronal firing rate during amygdala stimulation was calculated and subjected to two-way ANOVA (SigmaStat, version 3.5), with the factors treatment (vehicle, AF, FAT mixture, diazepam, and fluoxetine) and type of response (↑ cells, ↓ cells, and *Ø* cells), followed by the SNK *post hoc* test. Values of *P* ≤ 0.05 were considered statistically significant. The data are expressed as mean ± standard error of the mean.

## 3. Results

### 3.1. Histological Control

**Figure 1:**
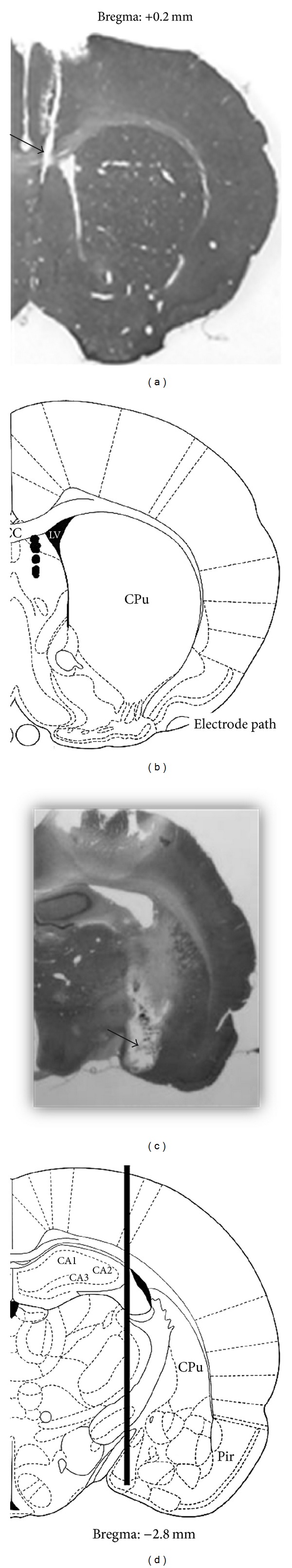
Location of electrodes. (a) Coronal section of the brain (Nissl technique) that shows the recording electrode tips in the dorsal aspect of the lateral septal nucleus (arrow). (b) Representative schematic of a coronal section (+0.2 mm) from a rat that illustrates the marks left by the recording electrode. (c) Coronal section (Nissl technique) that shows the marks left by the stimulation electrode in the medial amygdala (arrow). (d) Representative schematic of a coronal section (−2.8 mm) from a rat that illustrates the marks left by the stimulation electrode. CPu: striatum; LV: lateral ventricle; C: corpus callosum; Pir: piriform cortex.

A total of 267 single-unit extracellular recordings were obtained from the lateral septal nucleus. The distribution of the recordings included 56 neurons in the vehicle group (*n* = 12 rats), 59 neurons in the fluoxetine group (*n* = 12 rats), 52 neurons in the diazepam group (*n* = 11 rats), 53 neurons in the FAT mixture group (*n* = 13 rats), and 47 neurons in the human AF group (*n* = 12 rats). The histological analysis allowed us to determine that the neuronal recordings were obtained from the dorsal aspect (3.0–4.2 mm beneath the cerebral cortex) of the lateral septal nucleus (Figure 1). We did not find significant differences between the depth of recordings (range: from 3.6 ± 0.05 mm to 3.7 ± 0.04 mm below the cortex) among groups (*F*_4,262_ = 0.621, *P* = 0.648).

### 3.2. Lateral Septal Nucleus Firing Rate Prior to Amygdala Stimulation

**Figure 2:**
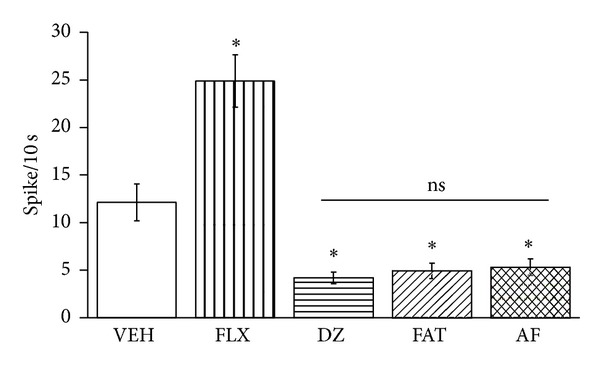
Overall effects of treatments. The lateral septal nucleus spontaneous neuronal firing rate is shown in the groups treated with vehicle (VEH), fluoxetine (Flx), diazepam (DZP), the fatty acid mixture (FAT), and fresh amniotic fluid (AF). The diazepam, fatty acid mixture, and fresh human amniotic fluid groups had the lowest values, and the fluoxetine group had the highest value compared with vehicle (*P* < 0.05, SNK).

The baseline lateral septal nucleus neuronal firing rate (Figure 2) was significantly different between treatment groups (*F*_4,262_ = 27.232, *P* < 0.001). The SNK *post hoc* test revealed that the highest (*P* < 0.05) firing rate was observed in the fluoxetine group and that the lowest (*P* < 0.05) firing rates were observed in the FAT mixture, human AF, and diazepam groups, with no significant difference between them. The septal neuronal firing rate in the vehicle group was intermediate.

### 3.3. Classification Based on Septal Response to Amygdala Stimulation

#### 3.3.1. Response Distribution

Table 1 shows the percent distribution of lateral septal neurons according to their type of response to electrical stimulation of the medial amygdala (base 1000 ms). Some variations were observed between groups in the distribution of the three types of responses.

### 3.4. Peristimulus Histograms

**Figure 3:**
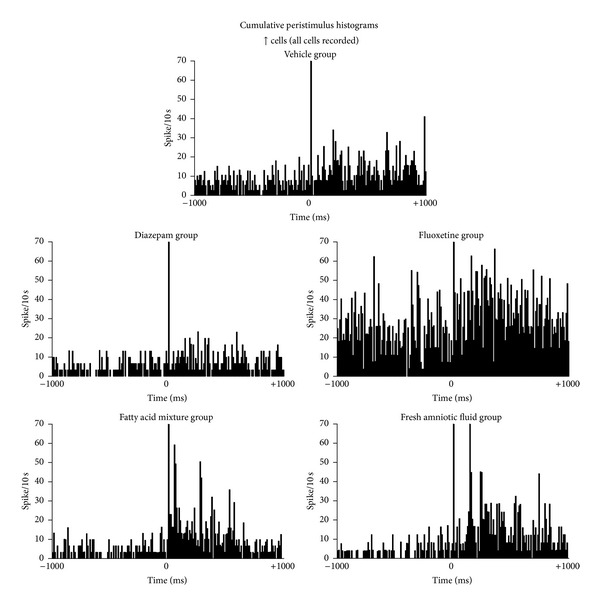
Cumulative peristimulus histograms. Septal neurons that responded with an increased firing rate (↑ cells) during amygdala stimulation are shown. Notice the qualitative change in firing in the FAT mixture and fresh human AF groups. Each histogram contains the mean firing rate (ordinate) from all recorded cells, 1000 ms before and 1000 ms after amygdala stimulation (abscissas).

Figure 3 illustrates the cumulative peristimulus histograms of all septal ↑ cells. The qualitative analysis indicated that diazepam (*n* = 18) reduced the septal firing rate compared with the vehicle group (*n* = 24). Fluoxetine (*n* = 19) produced the opposite action. In the period prior to amygdala stimulation, the septal neuronal firing rate was similar among the FAT mixture (*n* = 28), AF (*n* = 22), and diazepam (*n* = 18) groups. However, the increased firing rate in response to amygdala stimulation was higher in the AF and FAT mixture groups than in the vehicle group. 

**Figure 4:**
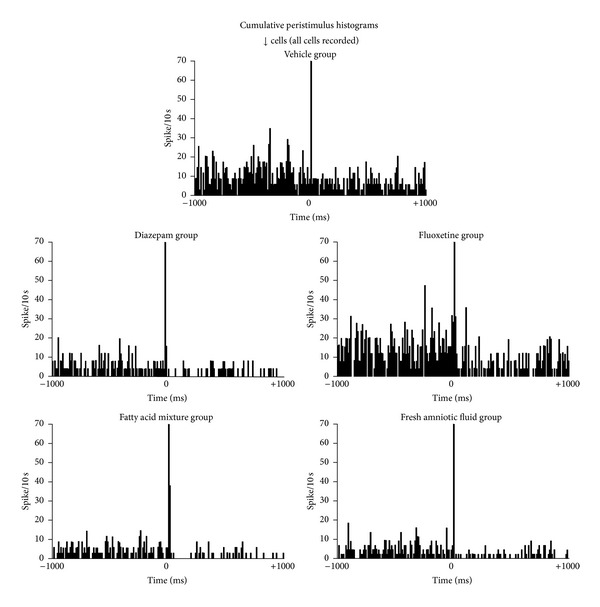
Cumulative peristimulus histogram for septal neurons that responded with a decreased firing rate after amygdala stimulation (↓ cells). The response was similar among treatments. The coordinates are the same as in Figure 3.

The cumulative peristimulus histogram of ↓ cells is shown in Figure 4. The qualitative analysis of the period prior to amygdala stimulation showed similarities between the vehicle (*n* = 12) and fluoxetine (*n* = 10) groups. The other three groups (diazepam, *n* = 14; FAT mixture, *n* = 9; AF, *n* = 15) fired at a similarly lower frequency.

**Figure 5:**
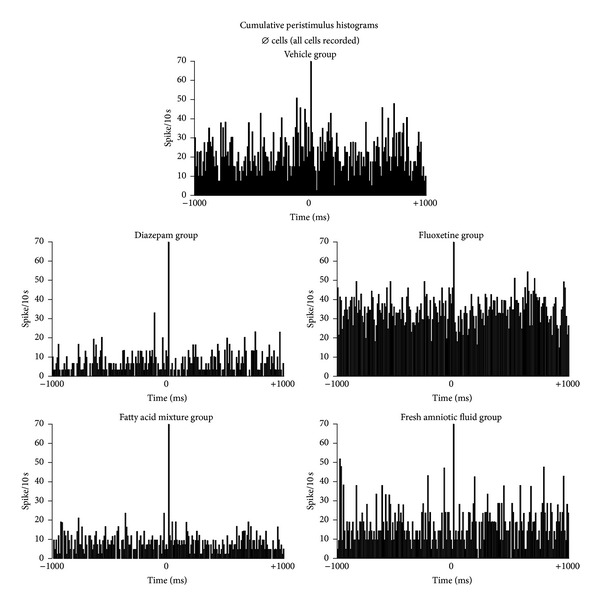
Cumulative peristimulus histogram for septal neurons that did not respond to amygdala stimulation (Ø cells). As expected, the differences were minimal and nonsignificant. The coordinates are the same as in Figure 3.

As expected, Ø cells did not respond to amygdala stimulation. The fluoxetine group (*n* = 30) qualitatively fired at the highest rate, whereas the diazepam (*n* = 20) and FAT mixture (*n* = 16) groups fired at the lowest rate. The vehicle (*n* = 20) and AF (*n* = 10) groups fired at similar frequencies (Figure 5).

#### 3.4.1. Percent Change in Firing Rate

**Figure 6:**
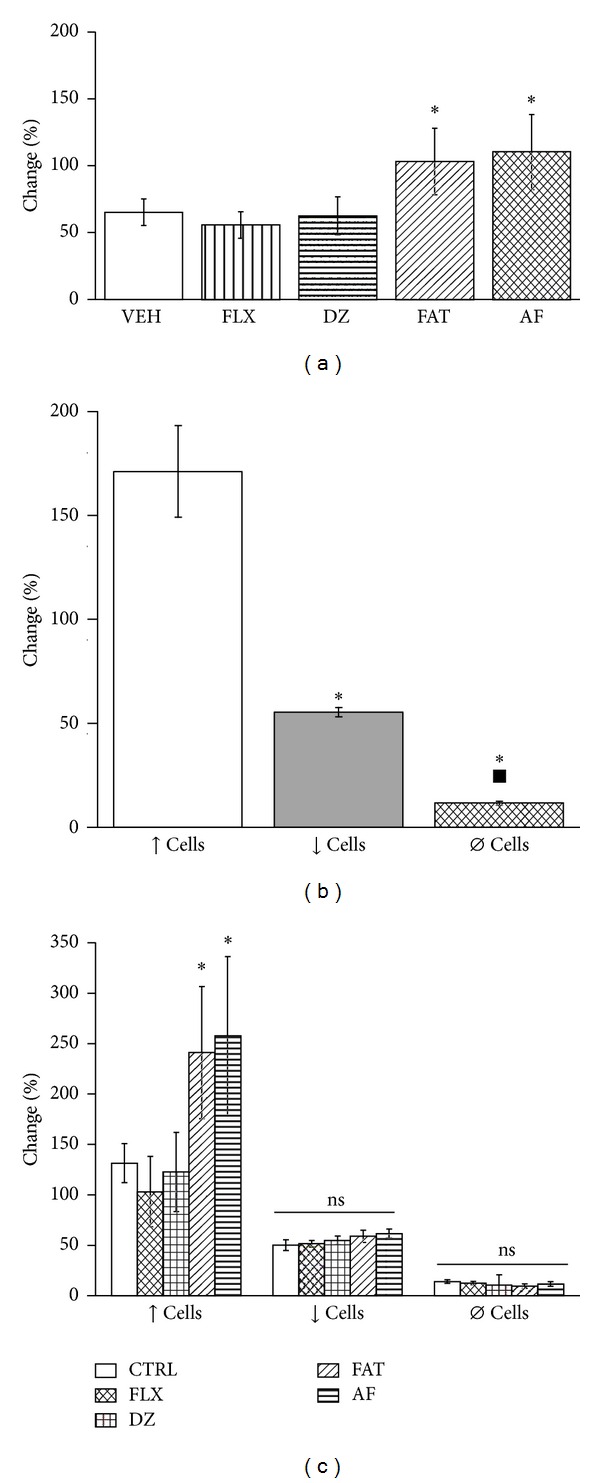
(a) Global percent change in the firing rate of lateral septal neurons in the different treatment groups. Only the FAT mixture and AF produced a significant change in firing rate after amygdala stimulation. (b) Percent change in neuronal firing rate in septal neurons based on the type of response. ↑ cells displayed the highest change in firing rate. (c) Only ↑ cells significantly changed their firing rate and only in the AF and FAT groups (*P ≤ 0.05, SNK). See Figure 2 for abbreviations.

The two-way ANOVA indicated a significant effect of treatment (*F*_4,262_ = 2.908, *P* < 0.02). The highest statistically significant (*P* ≤ 0.05, SNK; Figure 6(a)) change in firing rate was observed in the human AF and FAT mixture groups. Similar percent changes in septal neuronal firing rate were observed after amygdala stimulation in the vehicle, fluoxetine, and diazepam groups.

The analysis also indicated a significant effect of type of response (*F*_2,262_ = 55.424, *P* < 0.001). ↑ cells displayed the highest change in firing rate (*P* ≤ 0.05) compared with ↓ cells and *Ø* cells (Figure 6(b)).

The interaction between factors was also significant (*F*_8,262_ = 2.501, *P* < 0.01), and the *post hoc* analysis revealed that the change in the firing rate of septal neurons after amygdala stimulation reached statistical significance (*P* ≤ 0.05, SNK) only for ↑ cells and exclusively in the AF and FAT mixture groups and not in the other groups (Figure 6(c)).

## 4. Discussion

The present study investigated the effects of fresh human AF and its FATs on lateral septal neurons identified by their connection to the medial amygdala. Regardless of their response to medial amygdala stimulation, the FAT mixture and AF treatments decreased the spontaneous lateral septal neuronal firing rate similar to diazepam, whereas fluoxetine produced the inverse effect (i.e., an increased firing rate). An unexpected result was that the septal neurons that were most sensitive to AF or its FATs were those that responded with an increased firing rate after amygdala stimulation (↑ cells), an effect not observed with the other treatments.

The components used to prepare the FAT mixture correspond to intermediate-length FATs (from C6 to C18), and most of them are polyunsaturated [29]. Amniotic fluid is the natural environment for the development of the fetus [30], providing a safe environment [31] and seemingly exerting some anxiolytic/protective effects during early development, given the similar neural actions of AF, its FATs, and diazepam.

Fatty acids affect neuronal function by acting on the phospholipid membrane layer, modifying membrane tension, causing conformational changes in ion channels, and altering ionic conductance [32]. Fatty acids also exert neuronal effects, even after systemic administration [33], likely through a lipocalin superfamily [34] of protein transporters [15]. These proteins have also been identified in human AF [35], colostrum [36], and olfactory epithelia [37] that are connected to deep temporal lobe structures related to emotional processing [38], suggesting the existence of a complete receptor sensorial system for such maternal fluids.

In the present study, diazepam, AF, and its FATs decreased the firing rate of lateral septal neurons. Diazepam, through its actions on GABA_A_ receptors, produces hyperpolarization, with the participation of chloride channels [39]. Fatty acids produce mechanical actions on the conformation of GABA_A_ receptors [40], modifying the opening frequency of chloride channels [32, 41] and leading to neuronal hyperpolarization [32, 42]. This has been shown for oleic acid, one of the most abundant FATs found in AF [6, 16]. 

The spontaneous firing rate of lateral septal neurons decrease after a single forced swim session [43]. Several antidepressants produce an increase in firing rate [20, 44], which agrees with the present results. Such antidepressants exert their main actions on the serotonergic system [45], and we detected opposite actions between fluoxetine and diazepam, AF, and the FAT mixture, suggesting minimal participation of 5-HT in the effects of AF and its FATs. The actions of diazepam, AF, and its FATs were similar, suggesting the involvement of GABA_A_ receptors, a neurotransmission system already identified in the septal nucleus [46] and sensitive to treatments that reduce immobility in the forced swim test and increase the neuronal firing rate of septal neurons [20, 47], seemingly representing a neuronal correlate of the struggling behavior represented by the effort to solve an unsolvable problem.

An unexpected result arose from the cumulative peristimulus histogram analysis. Amygdala-septal neurons that responded with an increased firing rate (↑ cells) displayed a significant percent change only in the AF and FAT groups and not in the other groups. The amygdala complex contains several nuclei [48], and some specific functions of the medial amygdala have been identified. The lateral septal nucleus receives afferents from the medial amygdala [49], and this amygdala nucleus relays pheromonal and olfactory information to lateral septal neurons and participates in social behavior [50], which may be particularly relevant for maternal behavior. Our results support this possibility. Amniotic fluid is the maternal-fetal fluid in which the fetus develops and is in constant contact for months. Amniotic fluid and its FATs selectively produced a significant change in the firing rate of septal neurons that apparently receive excitatory inputs from the medial amygdala. 

The septal excitatory pathway comes exclusively from the medial amygdala [51] and is involved in disinhibition processes [52], with the participation of vasopressin and oxytocin [50, 53, 54]. Amniotic fluid and its FATs may promote the activity of these connections and impinge on filial behavior.

We used different routes of administration for every treatment included in present study. It may be argued that this fact may lead to some problem for the interpretation of results. However, in every case at least one hour elapsed between treatments and tests. Consequently, all used treatments must be reaching enough plasma concentrations during tests, as confirmed by differences found against vehicles.

## 5. Conclusion

Amniotic fluid and a mixture of its FATs produced diazepam-like effects on lateral septal neurons, regardless of their connection with the medial amygdala, likely with the participation of GABA_A_ receptors. Septal neurons receive excitatory inputs from the medial amygdala and appear to participate in the integration of maternal cue recognition mediated by AF and its FATs.

## Figures and Tables

**Table 1:** Lateral septal neuron distribution according to type of response to electrical stimulation of the medial amygdala.

	Control (*n* = 56)	Fluoxetine (*n* = 59)	Diazepam (*n* = 52)	Fatty acid mixture (*n* = 53)	Amniotic fluid (*n* = 47)
↑ Cells	42.8% (*n* = 24)	32.2% (*n* = 19)	34.6% (*n* = 18)	52.8% (*n* = 28)	46.8% (*n* = 22)
↓ Cells	21.4% (*n* = 12)	16.9% (*n* = 10)	26.9% (*n* = 14)	16.9% (*n* = 9)	31.9% (*n* = 15)
*Ø* Cells	35.7% (*n* = 20)	50.8% (*n* = 30)	38.4% (*n* = 20)	30.1% (*n* = 16)	21.2% (*n* = 10)

↑ Cells: cells with increased neuronal firing; *Ø* Cells: cells with no change in neuronal firing; ↓ Cells: cells with decreased neuronal firing; *n*: number of cells recorded.
